# Frontiers of parasitology research in the People's Republic of China: infection, diagnosis, protection and surveillance

**DOI:** 10.1186/1756-3305-5-221

**Published:** 2012-10-04

**Authors:** Jun-Hu Chen, Hen Wang, Jia-Xu Chen, Robert Bergquist, Marcel Tanner, Jürg Utzinger, Xiao-Nong Zhou

**Affiliations:** 1 National Institute of Parasitic Diseases, Chinese Center for Disease Control and Prevention, WHO Collaborating Centre for Malaria, Schistosomiasis and Filariasis, Key Laboratory of Parasite and Vector Biology, Ministry of Health, Shanghai, 200025, People’s Republic of China; 2 Department of Microbiology and Parasitology, Institute of Basic Medical Sciences, Chinese Academy of Medical Sciences and School of Basic Medicine, Peking Union Medical College, Beijing, 100005, People’s Republic of China; 3 Ingerod, 407, Brastad, Sweden; 4 Department of Epidemiology and Public Health, Swiss Tropical and Public Health Institute, P.O. Box, CH-4002, Basel, Switzerland; 5 University of Basel, P.O. Box, CH-4003, Basel, Switzerland

**Keywords:** Parasitology, Infection, Diagnosis, Surveillance, Public health responses, People’s Republic of China

## Abstract

Control and eventual elimination of human parasitic diseases in the People's Republic of China (P.R. China) requires novel approaches, particularly in the areas of diagnostics, mathematical modelling, monitoring, evaluation, surveillance and public health response. A comprehensive effort, involving the collaboration of 188 scientists (>85% from P.R. China) from 48 different institutions and universities (80% from P.R. China), covers this collection of 29 articles published in *Parasites & Vectors*. The research mainly stems from a research project entitled “Surveillance and diagnostic tools for major parasitic diseases in P.R. China” (grant no. 2008ZX10004-011) and highlights the frontiers of research in parasitology. The majority of articles in this thematic series deals with the most important parasitic diseases in P.R. China, emphasizing *Schistosoma japonicum*, *Plasmodium vivax* and *Clonorchis sinensis* plus some parasites of emerging importance such as *Angiostrongylus cantonensis*. Significant achievements have been made through the collaborative research programme in the following three fields: (i) development of strategies for the national control programme; (ii) updating the surveillance data of parasitic infections both in human and animals; and (iii) improvement of existing, and development of novel, diagnostic tools to detect parasitic infections. The progress is considerable and warrants broad validation efforts. Combined with the development of improved tools for diagnosis and surveillance, integrated and multi-pronged control strategies should now pave the way for elimination of parasitic diseases in P.R. China. Experiences and lessons learned can stimulate control and elimination efforts of parasitic diseases in other parts of the world.

## Review

### Background

The observed reduction of the transmission of several parasitic diseases in the world, particularly in the People’s Republic of China (P.R. China), has led to a rethinking of current approaches to control. In consequence, elimination of parasitic diseases is becoming a realistic option for many areas where transmission rates are falling and only relatively small pockets of transmission remain [1]. Such strategic changes entail a paradigm shift in research and public health response, i.e. instead of reducing only the burden of disease one can now attempt to interrupt transmission, which in turn implies a stronger role for rigorous surveillance approaches. Consequently, there are also a number of challenges with regards to research and development (R&D) for innovative or improved tools, e.g. drugs, vaccines, diagnostics and more precise delimitation of endemic areas based on remote sensing, geographical information systems (GIS) and geostatistical modelling [2,3].

In 2005, to cope with these challenges, the Chinese Science and Technology Major Programme, invested in a special research programme entitled “Surveillance and diagnostic tools for major parasitic diseases in P.R. China” (grant no. 2008ZX10004-011) that involved the collaboration of 188 scientists (>85% Chinese) from 48 different institutions and universities (>80% Chinese). The majority of articles published in this thematic series were funded by this special research programme that covers the major parasitic diseases in P.R. China, placing special emphasis on *Schistosoma japonicum*, *Plasmodium vivax* and *Clonorchis sinensis* and other parasites of emerging importance, such as *Angiostrongylus cantonensis* (Table 1).

**Table 1:** **Improved tools and strategies for control and eventual elimination of parasitic diseases in P.R. China that have been reviewed in a collection of papers published in *Parasites & Vectors***

**Diseases**	**Diagnosis**	**Surveillance**	**Strategies for public health action**
Schistosomiasis	Acute infection (CAs)	Google Earth	Integrated control or elimination strategy
	Immunological test	Mathematical modelling	
	Molecular test		
Malaria	Molecular test	GIS	Integrated control or elimination strategy
		Drug resistance	
		Genetic diversity	
Leishmaniasis	Immunological test	n.a.	n.a.
	Molecular test		
Toxoplasmosis	Immunological test	n.a.	n.a.
	Molecular test		
Clonorchiasis	Immunological test	n.a.	n.a.
	Molecular test		
Fascioliasis	Molecular test	Genetic diversity	n.a.
Angiostrongyliasis	Molecular test	n.a.	n.a.
Co-infection	Immunological test	n.a.	n.a.

Note: CAs (circulating antigens), GIS (geographical information system), n.a. (not available).

Considerable progress has been made through the collaborative special research programme with special reference to:

(i) development of integrated control strategies to strengthen national control programmes;

(ii) comprehensive update of the surveillance data on parasitic infections both in humans and animals; and

(iii) improvement of existing diagnostic tools or development of innovative tools to detect parasitic infections.

### A collection of articles in *Parasites & Vectors*

**Figure 1:**
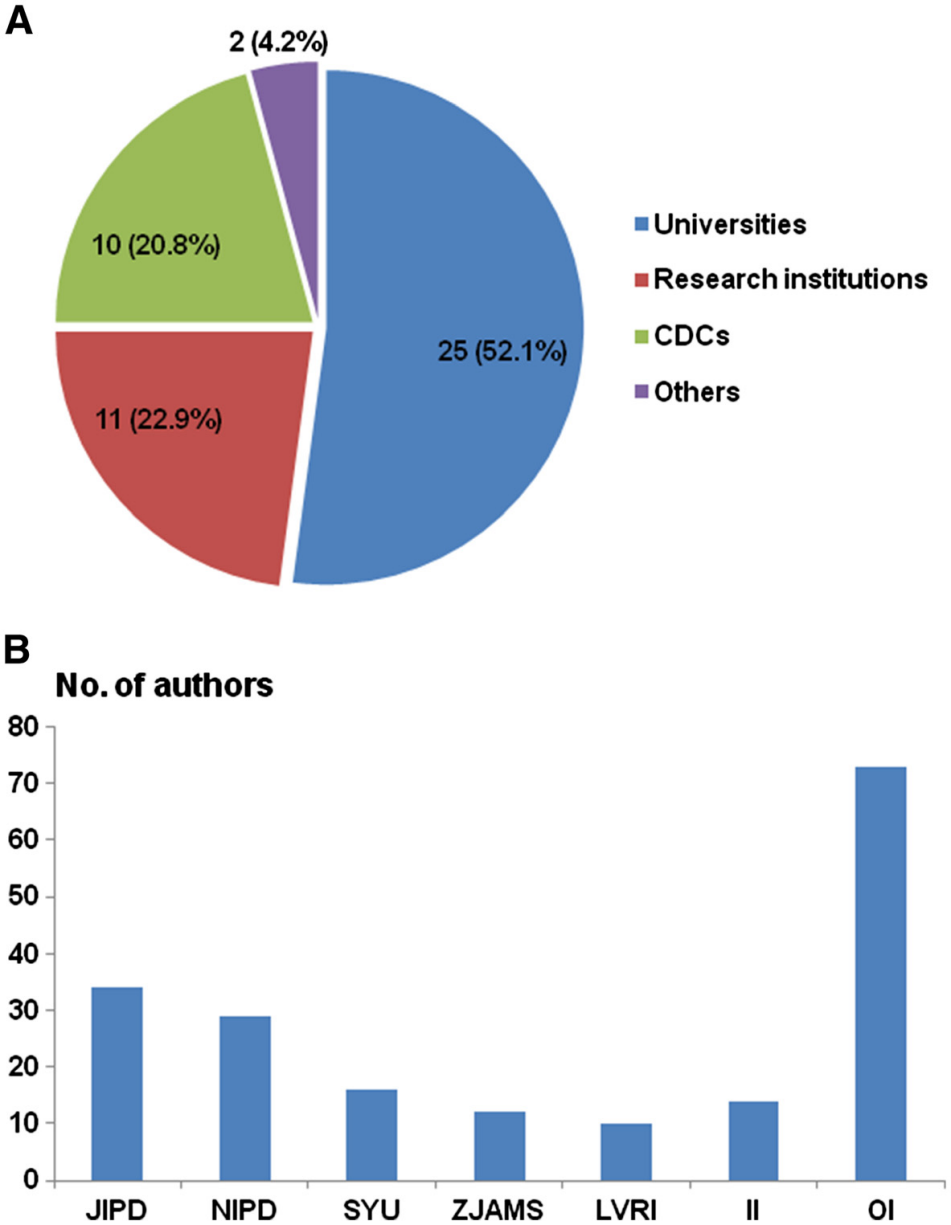
**Academic and research organizations that contributed to the 29 articles pertaining to various aspects of parasitology (e.g. surveillance, diagnosis, infection and immunity) in the People's Republic of China.** (**A**) Number and percentage (%) of contributing organizations. (**B**) Number of authors from each contributing organization (CDCs: institutions belonging to the Chinese Center for Disease Control and Prevention at different levels; NIPD: National Institute of Parasitic Diseases, China CDC; JIPD: Jiangsu Institute of Parasitic Diseases; SYU: Sun Yat-Sen University; ZJAM: Zhejiang Academy of Medical Sciences; LVRI: Lanzhou Veterinary Research Institute; II: international institutions; OI: other institutions).

The achievements reviewed here were reached through research collaboration and partnership among 188 researchers from 48 academic and research organizations (Table 2). More than half of the organizations (52.1%) are universities, 22.9% are research institutions, 20.8% different levels of organizations for disease control and prevention, and the remaining 4.2% are other research-oriented organizations (Figure 1A). The largest number of authors is affiliated with five organizations, namely (i) Jiangsu Institute of Parasitic Diseases (JIPD, n = 34); (ii) National Institute of Parasitic Diseases, Chinese Center for Disease Control and Prevention (NIPD/China CDC, n = 29); (iii) Sun Yat-Sen University (SYU, n = 16); (iv) Zhejiang Academy of Medical Sciences (ZJAMS, n = 12); and (v) Lanzhou Veterinary Research Institute, Chinese Academy of Agricultural Sciences (LVRI/CAAS, n = 10). A total of 14 international authors from 12 research institutions and other organizations abroad contributed to the special collection (Figure 1B).

**Table 2:** **Geographical analysis, stratifying the 148 authors contributing to this collection of 29 papers published in *Parasites & Vectors***

**Institution**	**Contribution of first author (number of articles)**	**Provenance of contributing authors**
National Institute of Parasitic Diseases, Chinese Center for Disease Control and Prevention	8	29
Jiangsu Institute of Parasitic Diseases	7	34
Zhejiang Academy of Medical Sciences	3	12
Lanzhou Veterinary Research Institute, Chinese Academy of Agricultural Sciences	3	10
Sun Yat-Sen University	2	16
Jilin University	1	8
China Medical University	1	7
China Agricultural University	1	5
Nanjing Medical University	1	5
Fudan University	1	3
Medical College of Soochow University	1	2
Anhui Center for Disease Control and Prevention	0	5
Anhui Institute of Parasitic Disease Control	0	5
South China Agricultural University	0	4
Bengbu Medical College	0	2
East China University of Science and Technology	0	2
Henan Center for Disease Control and Prevention	0	2
Jiangsu Institute of Nuclear Medicine	0	2
Jiangsu Provincial Department of Health	0	2
Peking Union Medical College	0	2
Sichuan Center for Disease Control and Prevention	0	2
Swiss Tropical and Public Health Institute	0	2
Tokyo Medical and Dental University	0	2
Center for Disease Control and Prevention of Jiuzhaigou County	0	1
Chinese Academy of Medical Sciences	0	1
Consejo Superior de Investigaciones Científicas	0	1
Dali Medical College	0	1
Dantu District Center for Disease Control and Prevention	0	1
Fuyang Center for Disease Control and Prevention	0	1
Gansu Agricultural University	0	1
Gifu University Graduate School of Medicine	0	1
Guangdong Institute for Animal Disease Control and Surveillance	0	1
Henan University of Science and Technology	0	1
Hubei Provincial Institute of Parasitic Diseases	0	1
Jiangxi Provincial Institute of Parasitic Diseases	0	1
Kangwon National University College of Medicine	0	1
London School of Hygiene and Tropical Medicine	0	1
Nagasaki University	0	1
Pennsylvania State University	0	1
University of Adelaide	0	1
University of Amsterdam	0	1
University of Nottingham	0	1
Wayne State University School of Medicine	0	1
Xinjiang Kizilsu Kirgiz Institute of Endemic Disease Prevention	0	1
Yangzhou Municipal Center for Disease Control and Prevention	0	1
Yongqiao District Center for Disease Control and Prevention	0	1
Yuangcheng Center for Disease Control and Prevention	0	1
Yunnan Agricultural University	0	1
48 institutions	29 articles	188 authors

**Figure 2:**
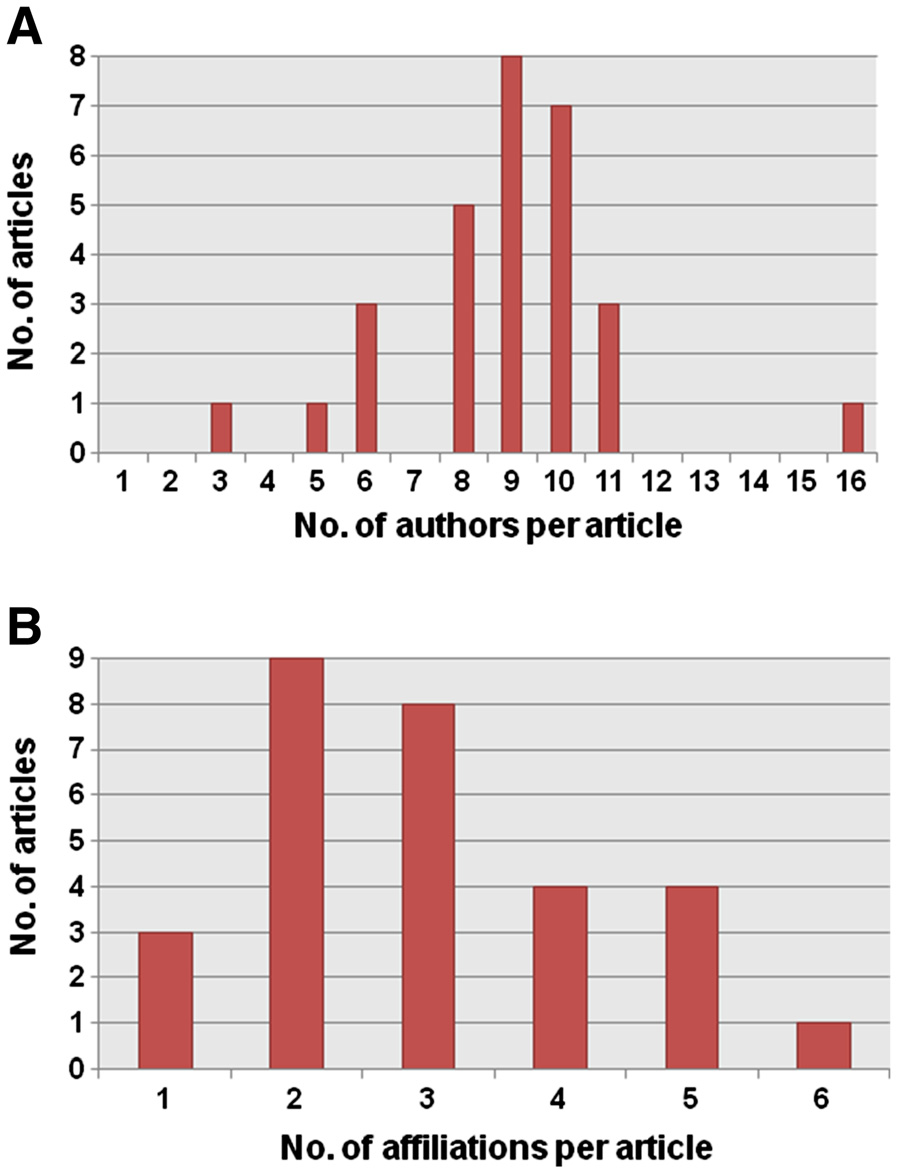
**Bibliometric analysis of the 29 articles published in the thematic series of “Aspects of parasitology in the People's Republic of China: surveillance, diagnosis, infection and immunity”.** (**A**) The number of articles according to the number of authors in each paper. (**B**) The number of articles according to the number of affiliations for each paper.

**Figure 3:**
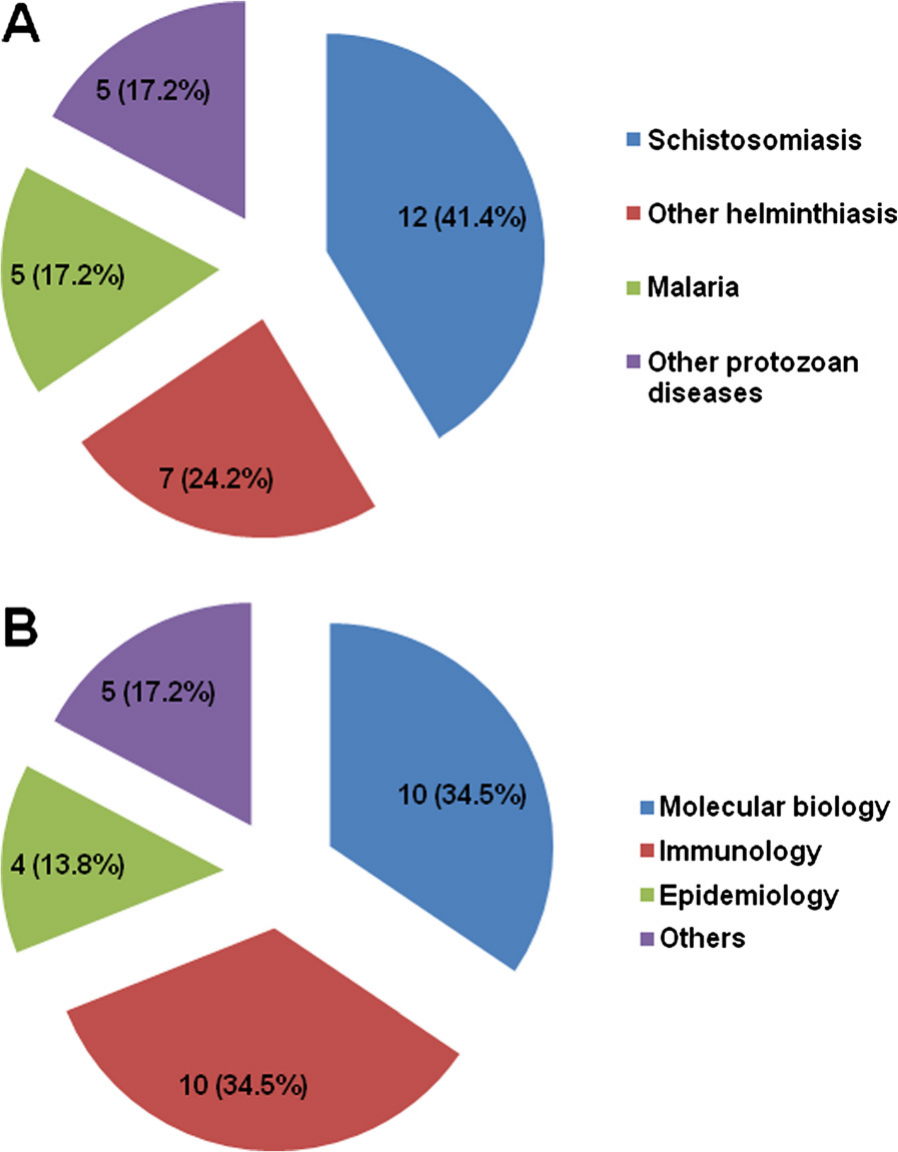
**Bibliometric analysis of the research topic and discipline for the 29 articles published in this thematic series of “Aspects of parasitology in the People's Republic of China: surveillance, diagnosis, infection and immunity”.** (**A**) Number and percentage (%) of articles according to disease research area. (**B**) Number and percentage (%) of articles according to the discipline involved.

The 29 papers featured in this collection of articles in *Parasites & Vectors* were each contributed by at least three and up to 16 authors and the number of affiliations for the authors in the individual paper ranged from one to six (Figures 2A and 2B), which indicates a strong and fruitful collaboration between academia and research organizations in the field of parasitic diseases in P.R. China. Schistosomiasis was the single most important parasitic disease covered (12 contributions, 41.4%), followed by malaria (17.2%). Other helminthiases and protozoal infections accounted for 24.2% and 17.2% of the articles, respectively (Figure 3A). The data thus underscore that schistosomiasis and malaria are still among the most important parasitic diseases in P.R. China. About a third of the articles belong to the disciplines of molecular biology and immunology (each 34.5%), whereas epidemiology was the main thrust in 13.8% of the articles (Figure 3B). Hence, the data reveal that improved tools based on molecular biology and immunology play important roles for the prevention, control and eventual elimination of parasitic diseases in P.R. China.

### Development of control and elimination strategies

An integrated control strategy for schistosomiasis japonica with emphasis on the reduction of infection sources has been put forward by Chinese scientists and consistently applied in many endemic areas since 2006. Colleagues from Jiangsu province reported impressive results by removing cattle from snail-infested grasslands, providing farmers with mechanized farm equipment, improving sanitation by supplying tap water and building lavatories and latrines as well as providing containers for faeces collection on fishermen’s boats. This strategy was implemented in 107 villages of the lower reaches of the Yangtze River and the effectiveness assessed during a 32-month period (May 2005 to January 2008) showing that it was possible to substantially reduce and even cut the transmission of *S. japonicum* by consistently implementing this integrated control strategy [4].

A study investigated the effect of a suspension concentrate of niclosamide (SCN) on *S. japonicum* cercaria, of which the new formulation of SCN is going to be used as emergency-treatment intervention for rapidly killing cercaria and eliminating water infectivity. This application of a well-known substance was shown to rapidly clear the risk of infection at specific water-contact sites for a limited time without unwanted adverse events, such as fish toxicity. It was thus concluded that this cercaria-killing method, as an emergency-treatment intervention for infested water, can be applied as part of a response package in forecasting and early warning systems (EWS) for schistosomiasis [5].

Despite the aforementioned progress, it is believed that achievements would be further consolidated with additional measures directed at *Oncomelania hupensis*, the intermediate host snail of *S. japonicum*. Yang *et al.*[6] provide an empirical framework for discerning the relative contribution of intrinsic effects (density feedback) from other extrinsic drivers of intermediate host snail population dynamics. They show that integrated schistosomiasis control measures must continue for a comparatively long time to reduce parasite abundance further because the snail populations tend to grow exponentially at low densities, especially *O. hupensis* populations in mountainous regions. It was concluded that density feedback in adult snail survival is the principal component contributing to the observed demographic phenomenon of population fitness (*r*)-abundance relationship, meaning that focal snail control needs to be taken into account, while implementing the new multi-pronged control strategy. Thus, a deeper understanding of *O. hupensis* density fluctuations is crucial if sustained reductions in snail numbers is to be achieved.

The South-to-North Water Transfer Project (SNWTP) is the largest national water conservancy project in P.R. China. Importantly, the Eastern Route Project (ERP) of SNWTP will cross the currently known habitats of *O. hupensis*. A study aimed at investigating the effects of the factors relating to the water diversion pattern on the spread northward of *O. hupensi*s and the changing risk of transmission of *S. japonicum* indicates that during the construction of the ERP of SNWTP, the risk of northward spread of schistosomiasis japonica will be decreased or eliminated as long as long-term reliable interventions for snail control are implemented [7].

### Surveillance of parasitic infections

The Government of P.R. China remains strongly committed to the consolidation of the achievements made over the past 50 years through the national schistosomiasis control programme in the country. Rigorous surveillance and immediate response at any sign of resurgence or re-introduction of the disease transmission in areas where the disease has been declared eliminated is critical for sustained success and is therefore an important feature of the national control programme. In controlled areas, the emphasis is on monitoring active infections among local residents and domestic animals (mainly water buffaloes), as well as monitoring suspected snail infestations, while the situation is carefully followed at sentinel sites located in the known endemic areas [8]. Sun *et al.*[9] imported Google Earth technology and a global positioning system (GPS) approach into the monitoring system for schistosomiasis surveillance of the banks of the Yangtze River in Jiangsu province. The results confirmed that the surveillance system can be rapidly updated and easily maintained, which proves that the Google Earth approach is a user-friendly, inexpensive EWS for schistosomiasis risk.

The food-borne trematodiases are an important group of neglected tropical diseases (NTDs) [10]. Infections with the opisthorchid liver flukes, e.g. *C. sinensis*, *Opisthorchis viverrini* and *O. felineus,* cause severe health problems globally, particularly in Southeast Asia [11,12]. The investigation of *C. sinensis* infection in its reservoir host dogs and cats revealed a high prevalence in P.R. China’s subtropical Guangdong province, which provides relevant “base-line” data for conducting control strategies and measures against clonorchiasis [13].

Protozoan parasites that infect humans are an extremely diverse collection of organisms that spans much of the eukaryotic tree of life [14]. *Toxoplasma gondii* is an important zoonotic intracellular protozoon parasite and *T. gondii* oocysts in pet dogs can traverse the intestinal tract and finally be excreted in the faeces, posing a serious threat to human health, particularly in pregnant women and immunologically deficient people. The modified agglutination test (MAT) was used to determine the seroprevalence of *T. gondii* infection in pet dogs [15]. A high prevalence of *T. gondii* infection was found in pet dogs in Lanzhou, north-west China, which has implications for public health.

Visceral leishmaniasis (VL) is a severe vector-borne parasitic disease of humans and other mammals caused by protozoa of the *Leishmania donovani* complex. Serological antibody tests and polymerase chain reaction (PCR)-based approaches have been extensively used to investigate canine infection with *L. infantum*[16]. More than half of all dogs living in the endemic Jiuzhaigou county were shown to be infected by this parasite. Control measures, such as treatment or elimination of infected dogs, or prohibition of maintaining dogs, must be taken due to the role of these dogs in the transmission of the infection to vectors. The current epidemiological profile and characteristics of VL in P.R. China based on retrospectively reviewing of VL cases between 2005 and 2010 was studied by a passive surveillance system showing that the number of VL cases and endemic counties both increased in the period 2005–2010 in P.R. China [17]. Different types or sub-types of VL revealed very distinct epidemiological characteristics [18]. Therefore, different control measures must be taken that are readily adopted to be specific epidemiological settings.

A study focused on determination of risk of malaria transmission by analyzing the shortest straight line distance from households to water bodies. It was found that most malaria cases (74%) were recorded in households located within 60 m of water bodies, which implies that very close proximity of households (i.e. ≤60 m) is a key risk factor for malaria transmission. This observation filled and important gap in the spatio-temporal analysis of malaria transmission, particularly with regard to settings where *Anopheles sinensis* is the vector species [19]. In order to better understand the role of the vector in the transmission of malaria during outbreaks, the vector capacity of *An. sinensis* in Huanghuai valley of central P.R. China was investigated. The study suggested that *P. vivax* malaria outbreaks in Huanhuai valley is highly related to the enhancement in vector capacity of *An. sinensis* for *P. vivax*, which was attributed to the local residents' habits and the remarkable drop in the number of large livestock leading to disappearance of traditional biological barriers [20].

Co-infection of parasites and other pathogens is poorly understood, but due to higher morbidity caused by co-infections, it warrants focused attention. With the potential spread of the human immunodeficiency virus (HIV) among rural Chinese populations, there is ample scope for co-infections with intestinal parasite infections and there is growing concern about their effects [21]. A study featured in this collection of articles published in *Parasites & Vectors* suggests that HIV-positive individuals are more susceptible to co-infection with *Cryptosporidium* spp. than their HIV-negative counterparts, particularly younger males with poor personal hygiene, indicating a need for targeted hygiene promotion, surveillance and treatment of intestinal parasites [22].

### Improvement of existing and development of novel diagnostic tools

#### Molecular tests

The loop-mediated isothermal amplification (LAMP) test is a high-performance method for detecting DNA, which holds promise for use in molecular diagnosis of pathogens in the first line battle against infectious diseases, including helminths and protozoa. LAMP uses a set of primers that initiate large-scale nucleic acid synthesis by *Bst* DNA polymerase at isothermal conditions. Research is now focused on the identification of sensitive and specific diagnostic tests for early identification of schistosomal infection and evaluation of chemotherapy in epidemiological surveys in P.R. China [23]. The data presented here indicate that LAMP is suitable for the detection of early infection in high-risk groups of *S. japonicum*, such as migrants, travellers, military personnel and school-aged children. However, it is less suitable for assessing efficacy of chemotherapy in the early stages because of its high sensitivity.

*A. cantonensis* is a zoonotic parasite that causes eosinophilic meningitis in humans [24]. The most common source of infection with *A. cantonensis* is the consumption of raw or undercooked molluscs (e.g. snails and slugs) harbouring infectious third-stage larvae (L_3_). However, the parasite is difficult to identify within the snails. The purpose of this study was to develop a quick, simple molecular method to survey for *A. cantonensis* in intermediate host snails. LAMP is an appropriate diagnostic method for the routine identification of this parasite within its intermediate host snail *Pomacea canaliculata* because of its simplicity and the high sensitivity and specificity. Hence, LAMP holds promise as a useful monitoring tool for *A. cantonensis* in endemic regions [25].

*P. vivax* is the major cause of malaria outside Africa, mainly in Asia and the Americas [26]. In P.R. China, more than 90% of the total malaria cases are due to *P. vivax*. In the central part of P.R. China, the re-emergence of malaria was considerable in provinces along the Huanghuai River, especially in Anhui and Henan provinces. As candidate methods for field malaria diagnosis, several different primer sets targeting numerous genes have been developed. It has been claimed that the LAMP method can detect as few as 100 copies of DNA template in blood samples (equal to roughly five parasites/μl of blood). This sensitivity is notably higher than any currently known immunochromatography-based malaria rapid diagnostic test (RDT) as recommended by the World Health Organization (WHO) as part of the global malaria control strategy [27], including PCR). A visualized LAMP method was established by the addition of a microcrystalline wax-dye capsule containing the highly sensitive DNA fluorescence dye SYBR Green I to a normal LAMP reaction prior to the initiation of the reaction [28]. Although further validation is needed and indeed ongoing, we can conclude that this novel, cheap and quick visualized LAMP method is feasible for malaria diagnosis under resource-constrained field settings in rural parts of P.R. China. Another novel LAMP targeting the 529 bp repeat element (529 bp-LAMP) was established to detect *T. gondii* DNA in blood samples of experimental mice infected with tachyzoites of the RH strain. Due to its simplicity, high sensitivity and cost-effectiveness for common use, we suggest that this assay should be used as an early diagnostic tool for health control of toxoplasmosis [29].

Early identification of the infection is essential to provide timely and appropriate chemotherapy to patients. Multiplex ligation-dependent probe amplification (MLPA) is a simple, robust and fast method designed for simultaneous detection of specific genomic sequences targeting multiple mutations to amplify specific MLPA probes rather than target DNA. The flexibility and specificity make MLPA a potential tool for specific identification of infections by opisthorchid liver flukes in endemic areas. MLPA has been used for rapid and specific detection of single nucleotide acid differences between *C. sinensis*, *O. viverrini* and *O. felineus*[30]. The flexibility and specificity make MLPA a potential tool for specific identification of infections by opisthorchid liver flukes in endemic areas.

Liver flukes belonging to the genus *Fasciola* are among the causes of human food-borne parasitic diseases [10]. It is important to characterize this species and the disease it causes, which has resulted in substantial economic losses to the livestock industry and has been increasingly observed to be of risk for humans as well. Therefore, current phenotypic techniques fail to reflect the full extent of the diversity of *Fasciola* spp. In this respect, the use of molecular techniques to identify and differentiate various species of *Fasciola* spp. offers considerable advantages. The advent of a variety of molecular genetic techniques also provides a powerful method to elucidate many aspects of *Fasciola* biology, epidemiology and genetics. However, the discriminatory power of these molecular methods varies, as do the speed, ease of performance and cost. There is a definite need for the development of new methods to identify the mechanisms underpinning the origin and maintenance of genetic variation within and among *Fasciola* populations. The increasing application of current and new methods, such as PCR- and LAMP-based approaches, will no doubt yield a much improved understanding of *Fasciola* epidemiology and evolution as well as more effective means of parasite control [31].

Since it is difficult to monitor the susceptibility of *P. vivax* to antimalarial drugs by *in vitro* tests, molecular markers of drug resistance are useful tools for mapping the current and changing pattern of pyrimethamine resistance of *P. vivax* isolates. *P. vivax* isolates were collected from four different sites in central P.R. China, and the sequence of the entire *dhfr* domain was determined to investigate genetic variation in *P. vivax* dihydrofolate reductase [32]. This study suggested that *P. vivax* in central P.R. China may be relatively susceptible to pyrimethamine. It also highlights that genotyping in the *pvdhfr* genes remains a useful tool to monitor the emergence and spread of *P. vivax* pyrimethamine resistance. Thus, genetic markers of parasite isolates are a potentially important part of the arsenal.

Transmission-blocking vaccines (TBVs) have been considered an important strategy for disrupting the malaria transmission cycle, especially for *P. vivax* malaria, which undergoes gametocytogenesis earlier during infection. Pvs25 and Pvs28 are transmission-blocking vaccine candidates for *P. vivax* malaria. Assessment of genetic diversity of the vaccine candidates will provide necessary information for predicting the performance of vaccines, which will guide us during the development of malaria vaccines. Genetic analysis revealed limited genetic diversity of *pvs25* and *pvs28*, suggesting antigenic diversity may not be a particular problem for Sal I based TBVs in most *P. vivax*-endemic areas of P.R. China [33].

#### Immunological detection

At present, selective chemotherapy with praziquantel is one of the main strategies in P.R. China's national schistosomiasis control programme, and thus diagnosis of infected individuals is of central importance. Thus far, a simple, affordable, sensitive and specific assay for field diagnosis of schistosomiasis japonica is not available, and this poses a great barrier towards full control let alone elimination of the disease [34]. Hence, a search for a diagnostic approach, which delivers these characteristics, is essential and should be given high priority [35].

Recently, a rapid and simple test for the detection of human antibodies against *S. japonicum* (i.e. the dipstick dye immunoassay (DDIA)) has been made commercially available in P.R. China. This assay produces results within 5–10 min without any more specialized instrument than a micropipette. The performance of DDIA was compared with stool examination evaluating its accuracy as a primary approach for screening the population in seven villages of low endemicity for schistosomiasis japonica [36]. Using stool examination as reference standard, DDIA performed with a high overall sensitivity of 91.3% and also a high negative predictive value, with a mean value of 99.3%. Hence, it was concluded that DDIA is a sensitive, rapid, simple and portable diagnostic assay and can be used as a primary approach for screening schistosome infection in areas of low endemicity. However, more sensitive and specific confirmatory assays need to be developed and combined with DDIA for targeting chemotherapy accurately. In addition to DDIA, another rapid dipstick assay (with the same specificity) based on latex particles and immunochromatography (DLIA), has been introduced [37]. The results show that DLIA is a simple, rapid, convenient, sensitive and specific assay for the diagnosis of schistosomiasis japonica and is therefore very suitable for large-scale field applications and clinical detection.

In addition to the above-mentioned assays, an innovative test for the diagnosis of schistosomiasis, a time-resolved fluoroimmunoassay (TRFIA), has been developed for detecting the signal transduction protein 14-3-3, a circulating antigen of *S. japonicum*. The results demonstrate that it has the potential of becoming a useful diagnostic method for schistosomiasis [38]. EWS with respect to the risk of infection is an important preventive measure against schistosomiasis. The study published here demonstrates that antibody responses to the Sj23HD antigen could be monitored for early detection of schistosome infection in mice, especially by immunoblot, which demonstrated greater sensitivity and specificity than an enzyme-linked immunosorbent assay (ELISA) for detection Sj23HD antibodies [39].

The detection of schistosome circulating antigens (CAs) is an effective approach to discriminate between previous exposure and current infection. Anti-adult worm antigen (AWA) IgY was produced by immunization of the hyline hens with AWA. Then purified IgY was immobilized onto the resin as a capture antibody to immune-precipitate CAs from patients' serum samples. The precipitated proteins were separated by one-dimensional electrophoresis and analyzed by LC-MS/MS. Four proteins including protein BUD31 homolog, ribonuclease, SJCHGC06971 protein and SJCHGC04754 protein were identified among the CAs, which could be used for biomarkers of diagnostics [40].

Cathepsin cysteine proteases play multiple roles in the life cycle of parasites such as food uptake, immune invasion and pathogenesis, making them valuable targets for diagnostic assays, vaccines and drugs. To find out whether cathepsin B could be applied for serodiagnosis in *C. sinensis* infection, a gene encoding this enzyme in *C. sinensis* (*Cs*CB) was identified and investigated with regard to its diagnostic value [41]. It was revealed that secreted CsCB may play an important role in the biology of *C. sinensis* and could be a diagnostic candidate for helminthiases.

#### Cellular immunity

Protection from acute toxoplasmosis is known to be mediated by CD8+ T cells, but the *T. gondii* antigens and host genes required for eliciting protective immunity have been poorly defined. The *T. gondii* dense granule protein 6 (GRA6), recently proved to be highly immunogenic and produces full immune protection in *T. gondii-*infected BALB/c mice with an H-2L^d^ gene. The CD8+ T cell response of H-2L^d^ mice infected by the *T. gondii* strain seemed to target a single GRA6 peptide HF10-H-2Ld complex. The result supports the concept that the acquired immune response is the major histocompatibility complex (MHC) restricted. This study has a major implication for vaccine designs using a single antigen in a population with diverse MHC class I alleles [42].

Helminth infection may modulate the expression of Toll-like receptors (TLR) in dendritic cells (DCs) and modify the responsiveness of DCs to TLR ligands. This may regulate aberrant intestinal inflammation in humans with helminths and may thus help alleviate inflammation associated with human inflammatory bowel disease (IBD). Epidemiological and experimental data provide further evidence that reducing helminth infections increases the incidence rate of such autoimmune diseases. Fine control of inflammation in the TLR pathway is highly desirable for effective host defense. Thus, the use of antagonists of TLR-signaling and antagonists of their negative regulators from helminths or helminth products should be considered for the treatment of IBD [43].

## Concluding remarks

This collection of articles in *Parasite & Vectors* confirms that the investment made by the Chinese government through a special research programme is starting to pay off. A considerable number of new developments and improvements of existing tools are reported in the 29 papers briefly summarised here. Besides scientific progress, this investment has led to capacity building and facilitated the adjustment and in some cases redirection of the efforts to control the various parasites that still threaten public health in P.R. China and elsewhere [44].

The focus of the majority of developments lies in the field of diagnosis related to *S. japonicum* (Table 1; for schistosomiasis see 12 out of 29 articles). It is evident that stable and easily applicable tests for low technology settings with sufficient sensitivity are still urgently required [45]. Additionally, training in case management at the individual and community levels need further support. While the results reported are promising, there is still much research and targeted public health action required to reach the goal to interrupt transmission and finally to eliminate schistosomiasis and other parasitic diseases in P.R. China. Elimination of any disease implies surveillance-response approaches with rapid and highly sensitive diagnostics for the surveillance of the target populations [46]. The “gold standard” parasitological diagnosis for humans is critical for the estimation of endemicity, but the standard methods currently in use are not sensitive enough at the lower prevalence levels we are encountering [47]. Thus, novel approaches like the LAMP technology need to be developed further and validated – also with regard to cost-effectiveness to effectively contribute to the elimination approaches currently in use.

Techniques appropriate for the surveillance of human parasites infections are essential for solving many of the current public health challenges. Tools for effective surveillance were already applied in the field of schistosomiasis, malaria and fascioliasis, such as GIS and modelling tools for surveillance of *S. japonicum* and *Plasmodium* spp., molecular tests for genetic diversity and drug resistance monitoring. Moreover, combined with improved tools for diagnosis and surveillance and connected to effective response packages will lead to integrated, multi-pronged strategies for control and elimination of parasitic diseases in P.R. China [48]. Experiences reviewed here will be important for other countries that are moving from morbidity control towards transmission interruption and eventual elimination of parasitic diseases.

## Competing interests

The authors declare that they have no competing interests.

## Authors' contributions

Analyzed the data: JHC, XNZ. Wrote the paper: JHC, HW, JXC, RB, MT, JU, XNZ. All authors read and approved the final version of the manuscript.

## Financial support

The study was funded through the National Science & Technology Mayor Project (grant no. 2008ZX10004-011, 2012ZX10004-022). XNZ was supported by a Senior Personnel Grant in Public Health of Shanghai (GWH102012507).
